# Theory-Based Design and Development of a Socially Connected, Gamified Mobile App for Men About Breastfeeding (Milk Man)

**DOI:** 10.2196/mhealth.5652

**Published:** 2016-06-27

**Authors:** Becky K White, Annegret Martin, James A White, Sharyn K Burns, Bruce R Maycock, Roslyn C Giglia, Jane A Scott

**Affiliations:** ^1^ School of Public Health Curtin University Perth Australia; ^2^ Reach Health Promotion Innovations Perth Australia; ^3^ Collaboration for Evidence, Research and Impact in Public Health (CERIPH) Curtin University Perth Australia; ^4^ Telethon Kids Institute University of Western Australia Perth Australia

**Keywords:** mHealth, smartphone, mobile phone, app, breastfeeding, fathers, gamification, social connectivity

## Abstract

**Background:**

Despite evidence of the benefits of breastfeeding, <15% of Australian babies are exclusively breastfed to the recommended 6 months. The support of the father is one of the most important factors in breastfeeding success, and targeting breastfeeding interventions to the father has been a successful strategy in previous research. Mobile technology offers unique opportunities to engage and reach populations to enhance health literacy and healthy behavior.

**Objective:**

The objective of our study was to use previous research, formative evaluation, and behavior change theory to develop the first evidence-based breastfeeding app targeted at men. We designed the app to provide men with social support and information aiming to increase the support men can offer their breastfeeding partners.

**Methods:**

We used social cognitive theory to design and develop the Milk Man app through stages of formative research, testing, and iteration. We held focus groups with new and expectant fathers (n=18), as well as health professionals (n=16), and used qualitative data to inform the design and development of the app. We tested a prototype with fathers (n=4) via a think-aloud study and the completion of the Mobile Application Rating Scale (MARS).

**Results:**

Fathers and health professionals provided input through the focus groups that informed the app development. The think-aloud walkthroughs identified 6 areas of functionality and usability to be addressed, including the addition of a tutorial, increased size of text and icons, and greater personalization. Testers rated the app highly, and the average MARS score for the app was 4.3 out of 5.

**Conclusions:**

To our knowledge, Milk Man is the first breastfeeding app targeted specifically at men. The development of Milk Man followed a best practice approach, including the involvement of a multidisciplinary team and grounding in behavior change theory. It tested well with end users during development. Milk Man is currently being trialed as part of the Parent Infant Feeding Initiative (ACTRN12614000605695).

## Introduction

### Breastfeeding

Breastfeeding is universally recognized as the optimal way for babies to receive nutrition, and breastfeeding offers many well-documented health benefits for both mother and baby [1-4]. Despite concerted effort in policy, research, and community and hospital practice, breastfeeding rates in Australia at 6 months, and in particular rates of exclusive breastfeeding, remain low [5]. Breastfeeding initiation rates are generally good, with 96% of Australian women initiating breastfeeding. However, rates decline steadily thereafter, with only 15% of babies exclusively breastfed at 5 months [5].

### Targeting Fathers

The influence of the father has been identified as one of the most significant factors influencing the breastfeeding behavior of the mother [6-10]. Scott et al reported that a woman’s partner has an important influence on the mother’s decision to initiate and to continue breastfeeding [11]. These findings were reinforced in 2015 with data from the Australian Infant Feeding Survey, which found that multiple factors have an impact on breastfeeding cessation, with the most influential factors being the partner’s views, the use of pacifiers, and maternal obesity [6].

Relatively few father-focused breastfeeding interventions have robustly evaluated breastfeeding outcomes using a randomized controlled trial (RCT) design. However, the Fathers Infant Feeding Initiative (FIFI), conducted by members of our team, trialed a male-facilitated antenatal class for expectant fathers and a follow up social support component consisting of age-relevant information being mailed out to participants [12]. The FIFI RCT reported a significant difference between intervention and control groups in the percentage of babies who received any breastmilk at 6 weeks of age (intervention: 81.6%, control: 75.2%) [12]. The researchers recommended extending the study to 6 months and separating the social support intervention from the male facilitator-led antenatal sessions to measure the relative effect. The study also reported that fathers expressed a preference for Internet, email, and video to be used as a basis for the delivery of information [13].

Mothers have reported that partner support makes a difference to their confidence, as well as helping them to achieve their breastfeeding goals [14,15], and fathers typically indicate they are supportive of breastfeeding and want to be involved [13,14,16,17]. Involving fathers and increasing their support for breastfeeding has been recommended repeatedly in the literature [10,11,16-19]. However, despite fathers generally being supportive of breastfeeding, the literature highlights several factors that can affect the level of support they are equipped to offer. These factors include social support, knowledge, empowerment, and other specific barriers (see Textbox 1 [13,14,16,17,19-29]).

**Factors affecting the support fathers offer to their breastfeeding partners.**

**Social support** [13,14,16,20-23]

- Insufficient social support
- Frequent exclusion from family support programs
- Lack of opportunities to learn and share
- Lack of peer support

**Gaps in knowledge** [13,14,16,17,19,22,24,25]

- Expectations about breastfeeding, bonding with baby, and about how life changes after baby arrives
- Health and other benefits of breastfeeding
- Practical suggestions to help family
- Professional services available, for mothers and fathers

**Empowerment** [14,16,19,20,22,26]

- Lack of recognition of paternal role
- Lack of understanding of importance of paternal support for breastfeeding
- Need for more information and practical advice on how men can better support their family

**Barriers** [14,16,17,22,23,26-29]

- Concerns around having to postpone bonding with baby until breastfeeding has finished, or around other ways to bond with baby besides feeding
- Public breastfeeding
- Feeling left out of the relationship (with their partner and with the baby)

### Mobile Technology and Health Promotion

While specific recommendations from the FIFI study focused on the use of the Internet and DVDs, the technological landscape has changed markedly since the FIFI study was implemented in 2008. Smartphone usage is now virtually ubiquitous in Australia. In July 2014, Deloitte estimated that 81% of Australians aged 14 years and over owned a smartphone [30]. App usage is also prevalent, with the Australian Communications and Media Authority finding that 75% of Australian smartphone users had downloaded an app to their smartphone in a 6-month period [31]. Data from the United States in 2014 revealed that Android and iOS smartphone users were spending 65% more time using apps than they had 2 years previously, equating to 30 hours and 15 minutes each month per user [32]. Australians now spend more time accessing the Internet from smartphones than they do from desktop computers [33].

Mobile technology has been incorporated into health promotion programs targeting various health behaviors. Initiatives have targeted new parents [34], physical activity and nutrition [35-37], alcohol [38], suicide prevention [39], and mental health [40,41]. The use of smartphones offers specific benefits in terms of high user engagement [42], including the opportunity to deliver ecological momentary interventions [43]. These are interventions that occur as people participate in their daily lives and happen in real time [43]. As mobile users become increasingly savvy about app usage [44], their expectations grow, and it is important that apps developed for research purposes match the usability and sophistication that users expect from other “real-world” apps.

Developing mHealth interventions in multidisciplinary teams is a best practice approach recommended by many researchers [45-47]. It is important to design apps that are a good fit for user expectations and that make effective use of the devices on which they are deployed. Working with app development professionals early in the process can help to ensure that apps are well planned and executed [48]. This involvement can also identify trends in app development and user behavior, which may be incorporated into an app-based health intervention. In the case of Milk Man, we included push notifications, social connectivity, and gamification as engagement and motivational strategies.

#### Push Notifications

Push notifications are a means by which mobile apps can send information or alerts to users [49]. Compared with other notification methods, such as email, push notifications are immediate and quick to act upon; swiping the notification takes the users directly to the app, and even into the specific context referenced by the notification. Notifications remain in a list until they are acted upon or removed, meaning they can potentially act as triggers for later action. Use of push notifications means that the onus is not solely on a participant to remember to engage with the service; to some extent the service comes to them.

#### Social Connectivity

The use of technology for information gathering has changed markedly over the last 20 years. Increasingly, people want to interact with technology and use it to socially connect rather than simply passively receiving static information [50]. Many people are now socially connected throughout the day, over a number of platforms. Australians are enthusiastic users of social media, with approximately 68% of Internet users having at least one social media profile [51]. Breastfeeding research with fathers shows that peer support and peer connection is highly valued [14,16,21,23], and results from the FIFI study demonstrated that this approach can affect women’s breastfeeding duration [12].

Socially connected mobile technology can encourage people to reach out to each other and build communities [52-55]. Encouraging results have been reported in studies of online social support communities in interventions across a broad spectrum of health areas, including weight management [52,56], physical activity [57], and social anxiety [58]. For example, a focus group study that investigated the feasibility of an app for overweight adults suggested that social support networks that create a virtual community could be the primary component in creating a successful healthy lifestyle app [52].

#### Gamification

Gamification is the practice of using game-like components to motivate and encourage people in non-game contexts, and it is becoming increasingly popular in health and fitness apps [59]. Gamification elements include badges, leaderboards, points, and challenges [60]. Evidence about the increasing use of gamified apps in health is emerging [47,61,62]. A review of physical activity and nutrition apps found that the use of gamification was widespread; however, behavior change theory was not widely incorporated and there was no industry standard for developers [62]. Several studies have noted the need for further investigation of the potential for gamified health apps to effect behavior change [47,61-63]. Australian mental health research with young men suggested that gamification may be of value in enhancing engagement and enjoyment with using technology [64].

### Parent Infant Feeding Initiative

We developed the Milk Man app to be trialed as part of the Parent Infant Feeding Initiative (PIFI), which has been previously described [65] (ACTRN12614000605695). The PIFI study is a 4-armed RCT comprising 1 control group, 2 medium-intensity intervention groups, and 1 high-intensity intervention group. Participants are being recruited from antenatal classes at hospital sites in metropolitan Perth, Western Australia. The control group has access to the usual care provided by the hospital. One medium-intensity group receives a male-facilitated antenatal class, while the other has access to the Milk Man app. The high-intensity group has access to both the male facilitator-led antenatal class and the Milk Man app.

One of the largest intervention breastfeeding studies to target male partners, the PIFI study will be conducted between 2015 and 2017 and is expected to provide valuable insights into infant feeding outcomes. This paper focuses on the Milk Man app, with particular emphasis on the formative research underpinning its design, development, and preliminary testing.

This research adds to the literature by describing the design and development of, to our knowledge, the first breastfeeding app targeted at men. The app uses carefully considered mobile strategies to engage men with an issue that is typically seen as the domain of women, and the results will add to the literature on mHealth and health promotion, particularly with respect to what works for targeting men with breastfeeding initiatives.

## Methods

### App Design

We developed the Milk Man app as a socially connected information and support resource for men. It is focused on breastfeeding and infant feeding, but includes broader information on topics including early parenting, being a supportive partner, and local service providers. It is based on evidence about the main factors affecting fathers’ support of their breastfeeding partners and is informed by social cognitive theory (SCT). As part of the formative research process, we completed a marketing audit of current advertising campaigns investigating how health messages, products, and services were being designed for the target group. This information helped guide the design of the app. Breastfeeding interventions are often targeted at the mother, resulting in fathers reportedly feeling excluded from family support programs [14,16,19]. Milk Man was explicitly designed for, and targeted toward, fathers, and this was a key consideration in encouraging men to access and use the information.

**Figure 1:**
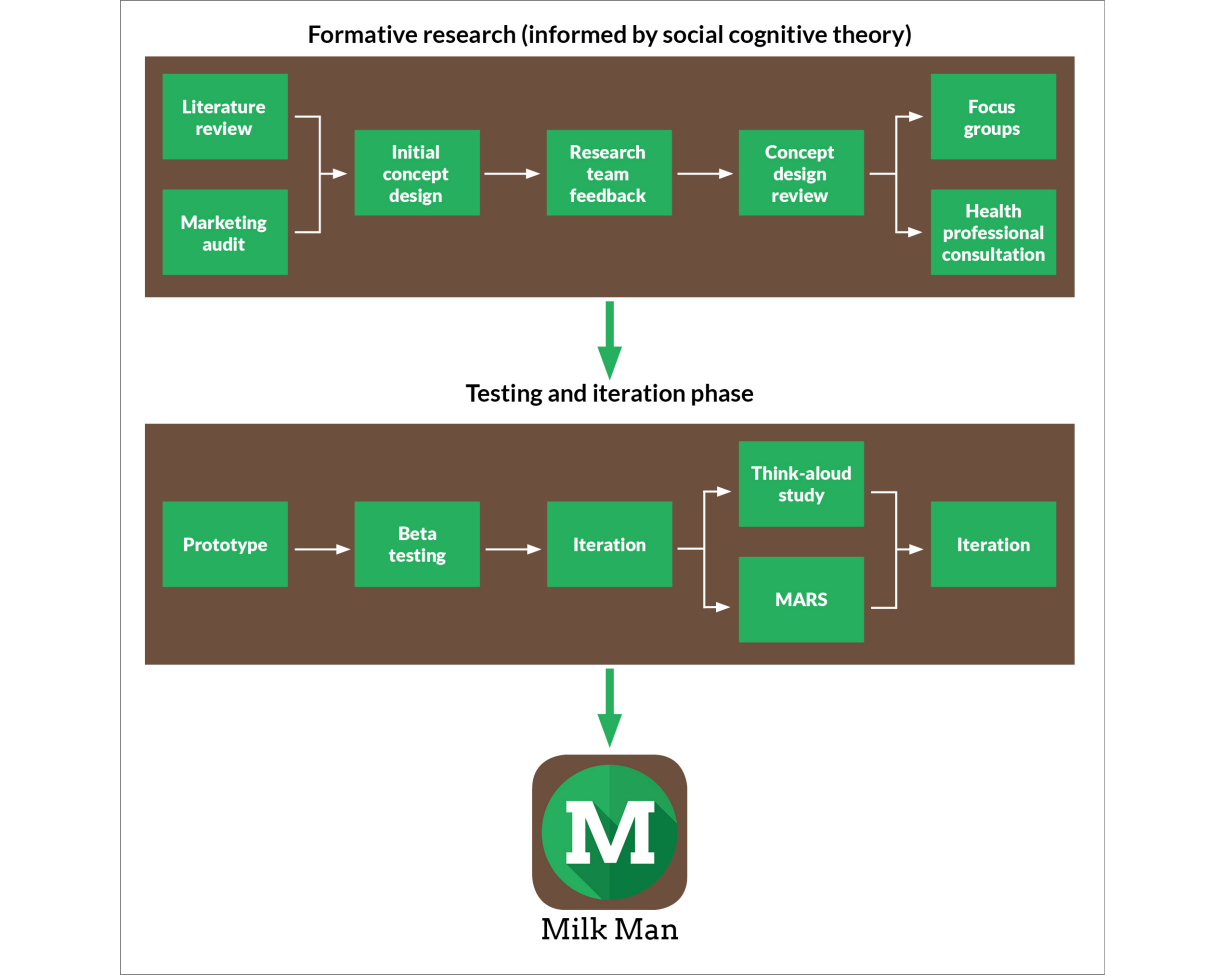
Development process for Milk Man.

Milk Man was informed by focus groups with men in the target group, in addition to consultation with health professionals. We refined it through a testing phase comprising beta testing and user testing with men in the target group. User testing involved participants completing a think-aloud walkthrough, as well as completing the Mobile Application Rating Scale (MARS) [66] (see Testing and Iteration sections below). Figure 1 illustrates the Milk Man development process.

### Theoretical Framework

SCT is a social learning model that operates at the interpersonal level, assuming an interaction between the social environment, the psychosocial determinants of behavior, and the individual [67,68]. In seeking to understand and predict human behavior, SCT can help to inform strategies for interventions to motivate and enable people to adopt healthier behaviors [69,70].

Reciprocal determinism is a key principle of SCT, describing the influence of both personal factors and the social environment on a person’s behavior. The factors that affect fathers’ decisions about and capacity to support breastfeeding are broad and include a combination of environmental and personal influences. Two specific social environmental factors that have been identified in the literature for this target group are the sometimes complex issues related to public breastfeeding, and the role that health professionals can have [16]. SCT acknowledges the impact these influences can have rather than simply focusing on the individual. In recognition of this, SCT has been recommended in the literature as a useful framework for breastfeeding interventions that target fathers [22,71]. It was used as the basis for the FIFI study, particularly in designing the male-facilitated antenatal sessions, which considered the constructs of self-efficacy and observational learning. It also helped researchers to understand the potential interrelation of different factors, including the overestimation of parental capacity and the underestimation of potential problems with breastfeeding.

We based the design of the Milk Man app and its engagement model on SCT constructs, to address the key issues affecting men’s support for their breastfeeding partners. The specific constructs of observational learning and goal setting were key components. In seeking to address self-efficacy, the app encourages problem solving between couples. Table 1 describes the theoretical framework underpinning the app and how the key engagement techniques used address the key factors identified in the literature.

**Table 1:** Milk Man engagement techniques mapped to social cognitive theory (SCT).

Key factors		SCT constructs	Engagement technique in Milk Man app
**Social support**			
	Men feel they do not receive enough social support with pregnancy and early parenting.	Observational learning Goal setting Self-efficacy	Connected social support function via the guided “conversation” feature. App was specifically designed for, and targeted towards men. Gamification functions to encourage inclusion, engagement, and positive feedback.
**Knowledge**			
	Men have gaps in knowledge around breastfeeding, pregnancy, and early parenthood.	Outcome expectations Goal setting Self-efficacy	Provision of information via the library, including practical solutions and support service contact details. Regular, age-relevant topics sent out as push notifications.
**Empowerment**			
	Men report lack of recognition of paternal role and understanding of their supportive role.	Self-efficacy Self-regulation Outcome expectations	Focus on empowering men to understand their role through the library and the conversation. Provision of practical advice Encouragement to discuss issues with partner.
**Barriers**			
	Men report specific barriers, including bonding postponement, public breastfeeding, and feeling left out.	Self-regulation Self-efficacy Observational learning Outcome expectations Goal setting	Forum for men to share information and an opportunity for discussion about solutions to barriers. Provision of information and strategies on public breastfeeding. Provision of information on specific barriers and solutions with the aim of establishing realistic outcome expectations.

### Engagement Strategies

We specifically designed the app to be attractive and engaging to the target group. The app is contemporary, delivers important information in a fun and lighthearted manner, and contains quirky imagery throughout. Milk Man contains engagement strategies that aim to keep men interested in using the app. The main engagement strategies are the use of push notifications, social connectivity via a guided conversation, an information library, and gamification.

#### Push Notifications

The Milk Man app has new content being added in the form of conversation topics twice a week. Push notifications are used to alert users to new discussion topics.

#### Social Connectivity Through Conversation

**Figure 2:**
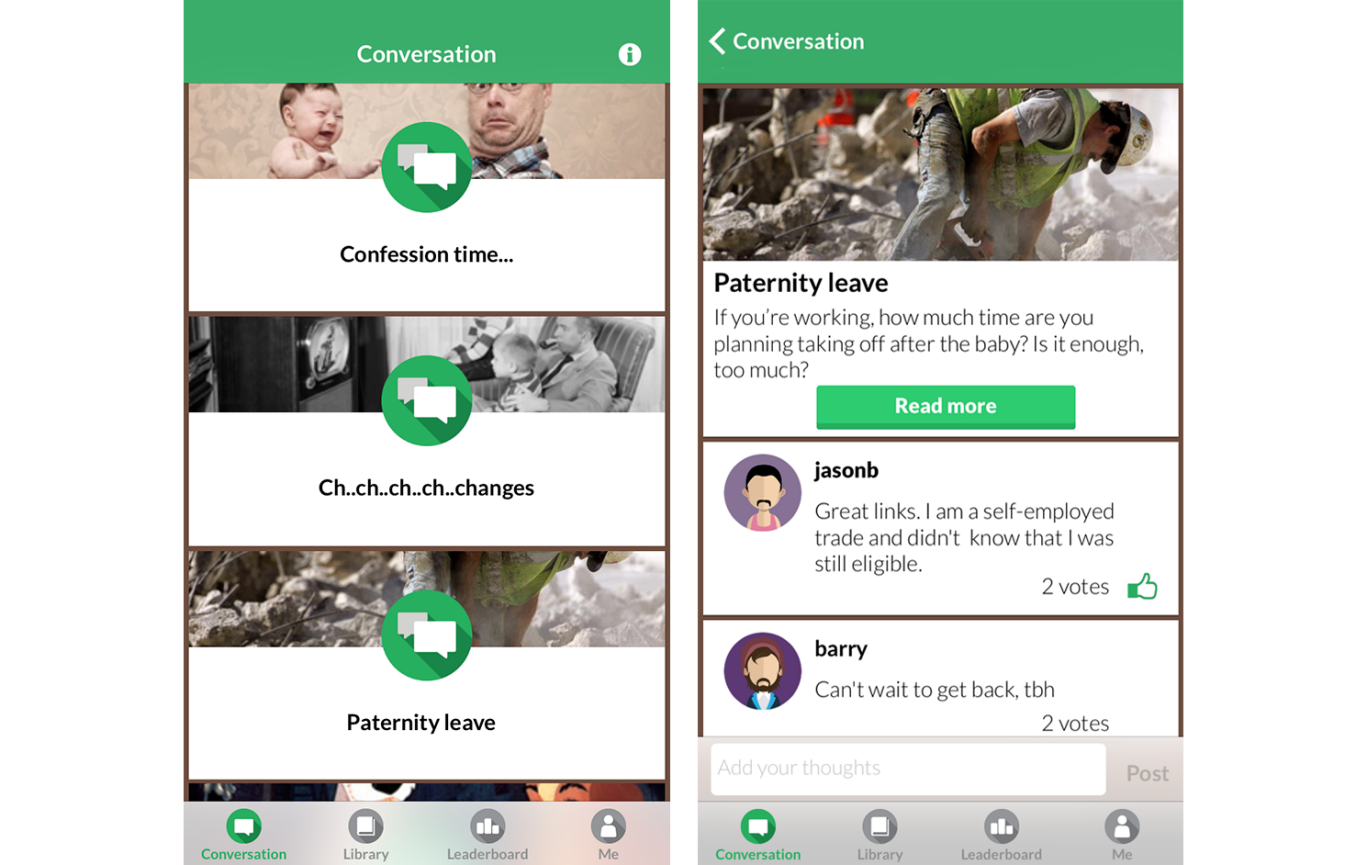
Milk Man conversation function.

Milk Man aims to socially connect men by engaging them in a guided conversation. The conversation consists of a series of topics initiated by the app administration team twice a week. Participants receive a push notification alerting them to new topics and inviting them to participate in the conversation. On swiping the notification, they are taken directly to that conversation within the app. Topics are either posts or polls. A post, shown in Figure 2, consists of a question, usually with a link to a static information article in the library component of the app.

Users can add comments to the conversation, and “upvote” (that is, like or recommend) other users’ comments. A poll is a multiple choice question, where users can choose an answer and view the aggregated responses of other users. Users are placed into conversation groups on the basis of the estimated due date of their baby, enabling age-relevant information to be sent at appropriate times.

#### Information Library

**Figure 3:**
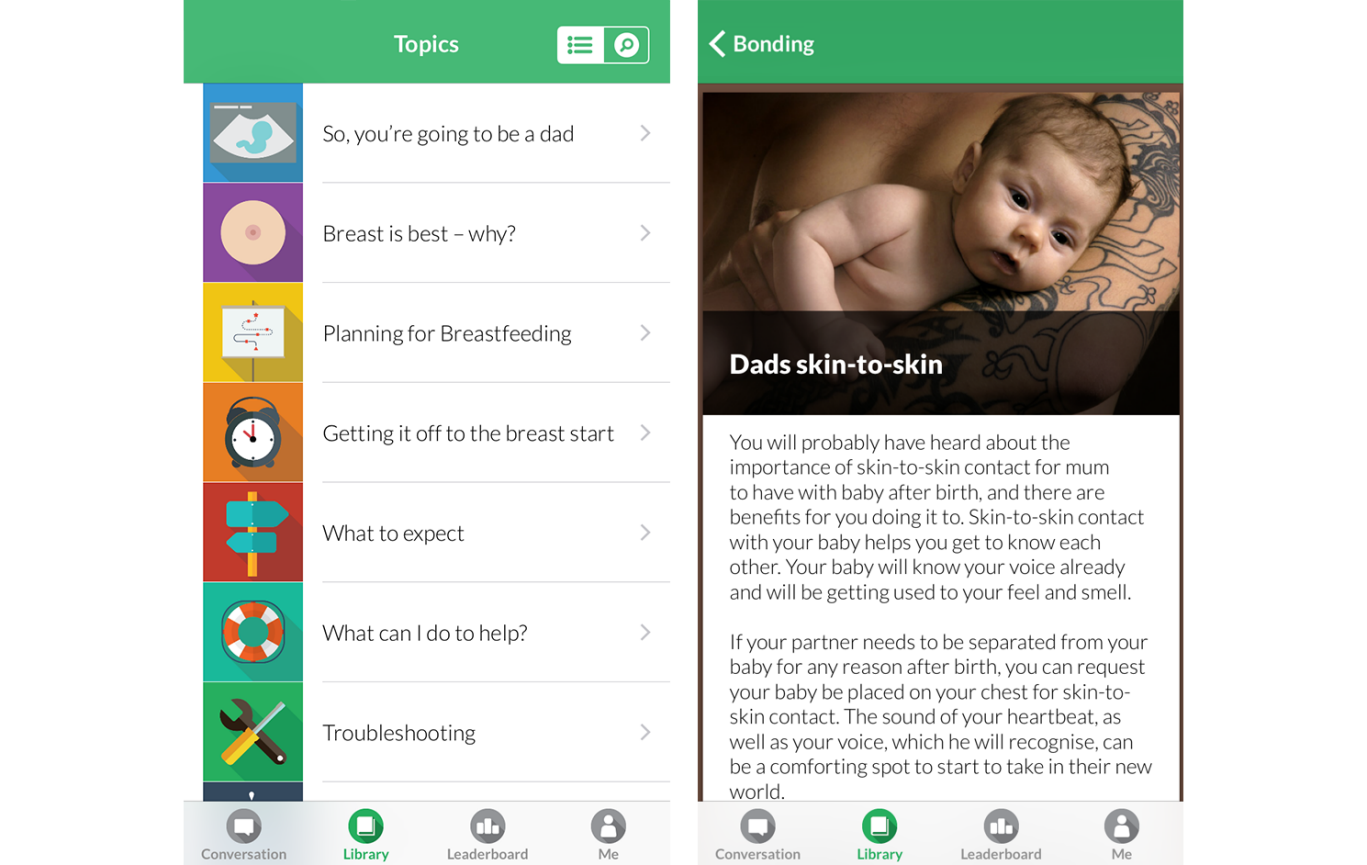
Milk Man library.

The app also contains a library of static, evidence-based information tailored specifically to fathers (see Figure 3). This includes information on preparing for fatherhood, breastfeeding and infant feeding, managing expectations, and how to seek support. The library uses the progressive disclosure technique [72], where information is sequenced so the initial information is concise, then progressively more detailed as the user requests further information. External links provide further information from service providers, including the Australian Breastfeeding Association [73] and the Raising Children Network [74]. We restricted the length of the articles to approximately 150 words to ensure content is succinct and minimal scrolling is required to see the whole article.

#### Gamification

**Figure 4:**
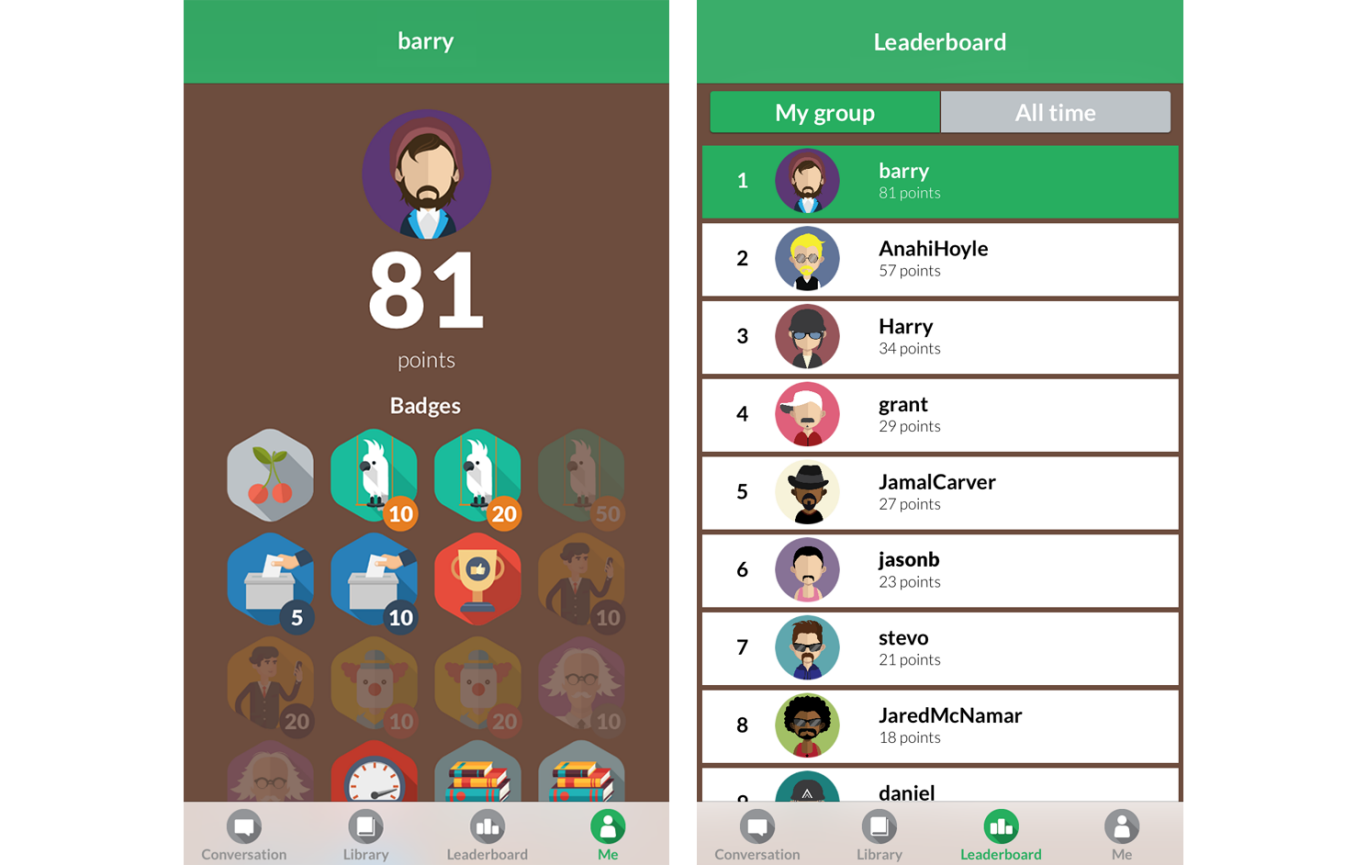
Gamification features used in Milk Man.

The app uses leaderboards, badges, and points to encourage engagement with both the social conversation and the static library of information. Users are awarded points for commenting on posts, contributing to the conversation, voting on polls, receiving upvotes from other users, and reading library articles. Users can see their score and rank on the leaderboard. Figure 4 shows these features.

### Formative Research to Inform Development of the App

#### Focus Groups With Target Group

Following internal review with the research team, we tested the initial app concept with members of the target group in a series of 3 focus groups. We recruited a purposive sample of men (n=18) through existing networks, including through the university staff and student body and a local playgroup. Participants were required either to be expecting a baby or to have a baby under the age of 6 months. The focus groups aimed to investigate the acceptability of the engagement strategies, provide guidance in the framing of the app, and ensure the proposed approach and content were appropriate. Participants were asked to complete a brief demographic survey before starting the session. The lead author recorded, transcribed, and reviewed the focus groups to maintain dependability [75].

#### Consulting Health Professionals

We held 2 separate consultative sessions with health professionals from 2 of the maternity hospital sites (1 public and 1 private) participating in the PIFI study. Before the session, we developed an outline of the library content to be included within the app, and this outline formed the basis for the discussion with stakeholders. We invited health professionals to comment on the proposed design, engagement strategies, and content of the app.

### Testing and Iteration of the App Prototype Phase

App testing was divided into 2 phases: beta testing and user testing. The beta testing involved providing early versions of the app to experienced app testers, who examined it for errors, crashes, layout issues, software bugs, or other problems. Beta testing was not carried out by members of the target group, as we were not seeking design and functionality feedback at this stage. Rather, it was tested by 4 experienced software testers, as well as members of the research team. We incorporated feedback into successive iterations of the app.

The second phase of testing, user testing, involved obtaining feedback on the app’s functionality, design, and usability. It was important that this phase of testing was carried out by members of the target group, as the objective was to gain an indication of the way in which the app was likely to be used and received by those for whom it was intended. Therefore, we invited participants to the testing phase if they were either expecting a baby or had a baby under the age of 6 months, and had previously expressed interest in the focus groups, but had not attended an earlier group. Once they had consented, we first asked participants to undertake a think-aloud walkthrough of the app, and then to complete the MARS [66,76]. We recruited 4 users to this testing phase. A previous study into think-aloud testing recommended that 4 to 5 test users is generally sufficient to identify up to 75% of usability issues, with the value of additional participants decreasing exponentially as the number increases [77].

#### Think-Aloud Walkthrough

Think-aloud walkthroughs are an industry standard approach in software development and a well-recognized way of testing mobile health apps [35,78-81]. In this study, after observing a researcher-led example using a different health app, participants were asked to spend a minimum of 10 minutes using Milk Man and to verbalize their thought processes as they navigated through the app. As the researcher wanted to observe the natural flow of app usage and observe organic navigation, the initial instruction was simply for users to “use and open the app as you would exploring any app for the first time.”

As the participants explored the app independently, the researcher monitored a checklist of 10 tasks and marked each off as it was completed. At the completion of the walkthrough, we specifically asked users to complete any tasks on the checklist that they had not completed unprompted. In keeping with best practice in conducting think-aloud studies, the researcher remained quiet throughout the study, speaking only to remind the participant to keep talking aloud and to issue tasks at the end. We recorded and transcribed the think-aloud sessions.

#### Mobile Application Rating Scale

Released in December 2014, the MARS is a comprehensive questionnaire used for rating mobile health apps with reference to 5 key criteria. The first 4 *objective* quality subscales give a measure of *aesthetics, engagement, functionality*, and *information*, while the fifth criterion is a *subjective* quality subscale and seeks users’ views on whether they would recommend the app, asks how often they would use it, and asks for an overall rating [66,76]. The MARS is scored by calculating the average of the 4 objective subscales. The MARS comprises 2 different versions, one for professionals, and a simplified version for app users. The app user version comprises 20 questions over the 5 criteria, with a final section asking 6 questions designed to describe the potential for impact on a user’s knowledge, attitudes, and intention to change [76]. After completing the think-aloud study, users were asked to independently complete the app user version of this scale.

## Results

### Focus Groups With Target Group

A total of 18 men attended the 3 focus groups. Participants were aged between 30 and 43 years. Most were married (n=14), just under half were expecting a baby (n=8), and just over half had a new baby aged under 6 months (n=10).

All men owned either an iPhone or Android smartphone, and all said that they kept their phone close at hand and referred to it throughout the day. All participants had some third-party apps on their smartphone. Most participants were positive about the idea of apps for new fathers. Most of the comments about the use of push notifications were positive, although some mentioned that they should be used judiciously and the content should be relevant.

I think the lesson really is notification fatigue. You know some people like them, some people don’t. I suppose if you got far too many you just become disinterested and that can actually be more dangerous than not getting a notification.

Focus group 1

There was a mixed response to the idea of a discussion forum for men to connect to each other. Some participants were very enthusiastic about the idea, while others stated they would not use it. Some of the reasons participants gave for ambivalence about a forum were not trusting the information, preferring to talk to people in real life, and that information on forums can be alarmist and cause unnecessary concern.

I don’t know, I wouldn’t talk to a stranger for starters on an app and then I mean you go, we go to barbeques and friends’ house and their kids are ratbags or this and that and you can’t tell your mate how to look after their kid, it’s their kid. You don’t know what they’ve been through the night before, you don’t know what they’ve eaten the night before, so I wouldn’t ask someone for advice on my child in that sense.

Focus group 3

We stopped trusting anything that wasn’t from a doctor ’cause we got 50 opinions and my wife ended up freaking out.

Focus group 1

Some participants suggested that humor and a lighthearted tone would be appropriate, and that the app should be quick and easy to use.

For me lighthearted would be better. Even the best baby I think that first period is probably strap in and get through it kind of time. So if I have to read a textbook of really...dry text I’m probably not going to do it. But if it’s something quick and easy that...tells me that what I’m seeing in front of me is correct [I’m more likely to use it].

Focus group 1

Push notifications can add that element to the humor.

Focus group 2

Reinforcing findings from the literature, men were also clear on wanting practical tips for helping their partner, with information ideally delivered in short, summarized formats, including bullets points and checklists. Access to more detailed information could be provided via links.

I want bullet points and if I want to read into it more I’ll look into it more if I’ve got the time.

Focus group 2

Checklists, perhaps a list of [reasons why] my baby won’t stop crying and then people could maybe leave suggestions. Doing an upvoting, downvoting vetted type system. Say “try this top answer, this worked really well” [or] “that didn’t work, give me another thing on the list to try.”

Focus group 1

Participants’ experiences with mobile apps were varied, as were responses to the proposed engagement strategies. Some participants had experience sourcing and using apps for parenting and pregnancy, while others identified specific barriers to their use, including issues with trusting information and preferring face-to-face interaction. In general, fathers supported targeting fathers with such an approach, and the focus groups provided good insights into how to structure the app’s engagement strategies.

### Consulting Health Professionals

To provide input about the content and engagement strategies proposed for the app, 16 health professionals attended 1 of 2 sessions. All participants were hospital-based midwives working with new and expectant parents. Some had additional, specialist roles: they were lactation consultants or parent educators, or they were in charge of discharge and follow-up of patients. Some specialized in working with aboriginal families, with young families, or with families requiring complex care relating to issues with alcohol and other drugs, or mental health problems.

The health professionals were generally enthusiastic about the app, and in particular about having men as the focus of the intervention.

Knowing the success of the woman’s breastfeeding experience is single-handedly influenced more by the support that [partners] give at home, than any other factor...makes [partners] feel like, “hey, I can do something to help.”

They want to help, but they don’t know how they can help.

The health professionals offered views that reinforced those from the focus groups, about keeping the tone of the app lighthearted, and ensuring the information provided was short and to the point.

Lighthearted and informative, because otherwise you’ll lose them, and they won’t come back if they’re finding it too heavy and judgmental.

Are you using dot form? Because I just find, they won’t read a whole big [article]. You just need dot points [and] keywords.

Pictures and dot points will work well.

Health professionals also offered specific content recommendations, including websites and online videos they typically used with new parents. They further advised the need to include information about postnatal depression for fathers and to focus on the message that every breastfeeding is a success.

### Testing and Iteration of App Prototype Phase

A total of 4 new or expectant fathers participated in the user testing phase. Of these 4 recruited participants, 3 had a baby aged under 6 months, while 1 was expecting a child. The age range was 34–44 years.

#### Think-Aloud Walkthrough

User testing via the think-aloud walkthrough identified 6 issues related to usability and functionality. Usability issues included text in the comments section being too small, a lack of clarity about how the points system worked, and the need for an important icon to be more prominent. In terms of functionality, 3 additional features were suggested: the ability for users to post their own questions, the inclusion of a tutorial or walkthrough to explain the different sections of the app, and the ability to later change the avatar they had selected on creating a user profile.

Most participants completed the 10 tasks on the walkthrough checklist while independently using the app, without needing to be prompted. In each case, they completed all of the remaining items when prompted.

#### Mobile Application Rating Scale

We averaged the MARS scores from each user list them in Table 2. All 4 participants said they would recommend the app, and they all gave the app a 4- or 5-star rating.

**Table 2:** Average (out of 5) Mobile Application Rating Scale (MARS) scores for each category applied to the Milk Man breastfeeding app.

MARS criterion	Average score
Aesthetics	4.3
Engagement	3.8
Functionality	4.6
Information	4.5
Total average score	4.3

## Discussion

### Developing and Refining Milk Man

Formative research was a critical component of the development process used for the Milk Man app. Guided by the existing literature, and theoretically underpinned by the SCT, the app content and functionality were refined and focused through feedback and input from clinical health professionals, members of the target group, and a multidisciplinary team of professionals. These professionals included breastfeeding researchers, health promotion professionals, nutritionists, and a midwife, as well as an app designer and developer. Qualitative data from the formative evaluative phase provided insight into the use of mobile technology by members of the target group and into what engagement strategies might be most effective. While this was not intended to be an exhaustive qualitative study to thematic saturation, there were many overlapping themes and participants provided rich insight to help guide the app development.

The testing phase identified 6 issues, 5 of which we addressed before starting the PIFI trial. The one identified issue that we did not act on was the suggestion that users could post their own conversation topics. We deemed this to be outside the scope of this research and a potential risk, in that topics could be poorly informed and contain inaccurate or misleading information. We added a brief tutorial (usually known as an onboarding exercise), to be displayed to users on first launching the app. This addressed several of the identified issues, including a description of the points system, an explanation of how the app worked, and an explanation of how users would be assigned to a group.

MARS scores were high, indicating good user acceptability, usability, and functionality. While still high, the engagement score was slightly lower. This appeared to relate to participants’ stated need for further instructions, explanations, and the ability to change avatars to better customize their user account, all issues that we addressed in the next iteration of the app.

### Next Steps for Milk Man

We have developed a comprehensive evaluation plan to measure the acceptability and effectiveness of the Milk Man app in the PIFI RCT. We will collect data through a mixed methods approach, including a customized analytics framework built into the app and a self-report questionnaire, which users will complete when their baby is 6 weeks old, and again at 26 weeks.

Evaluating adaptive technological interventions such as this requires a comprehensive approach, and we based the evaluation framework for this research on the one proposed by O’Grady et al [82]. This framework includes indicators for app users, content analysis, technology, computer-mediated interaction (user interaction with the interface), and broader health system integration.

While the use of mobile technology in public health interventions has grown significantly, there are still too few high-quality, adequately powered RCTs evaluating the use of such apps [83,84]. This large RCT will add to the evidence about the efficacy of mobile technology in delivering health interventions. The robust evaluation design will have broader relevance to public health interventions looking to use mobile technology to reach target groups.

### Limitations

This study sought to include the views of members of the target group in the app design and development through focus groups. Participants in the focus groups were aged between 30 and 43 years, meaning that younger fathers were not represented in this sample. This was due to the purposive sampling method used. However, this research builds on the aforementioned FIFI study, in which both younger and older fathers were consulted.

Although we recruited only 4 participants for the testing phase, this number has been previously shown to be effective in identifying most usability issues [77], and indeed participants’ reported issues overlapped significantly. While the MARS has been found to provide a reliable indicator of app quality when used by trained raters, the reliability of the app user version is being evaluated [66]. As such and because of the small number of users rating the app, these results should be interpreted with caution.

Technology changes quickly. There is a balance to be struck between developing health intervention apps in a thorough, methodical fashion and moving quickly to minimize the risks associated with a changing technological environment. To minimize these risks, we proceeded to the RCT without a pilot study. A larger pilot study of the app, before starting the PIFI study, would have been of value in providing further insight into the way in which men would use the app in a real setting. This may be particularly true of the more interactive components of the app, such as the conversation and the leaderboard; observing men engaging with these features may have further assisted refinement. However, we will be able to monitor this throughout and make those recommendations at the trial’s conclusion.

### Conclusion

Milk Man is a theoretically grounded app that provides information and support for the antenatal and postnatal periods and aims to socially connect fathers around a central theme of breastfeeding. We anticipate that providing a platform for men to discuss, share, and support each other through the breastfeeding journey will positively affect the support they offer their partners.

To our knowledge, Milk Man is the first breastfeeding app developed specifically for men. It uses innovative strategies to encourage user engagement. The development of Milk Man has involved stages of formative research, testing, and iteration. The process of design, development, and testing described here follows a best practice approach to app development, including being developed by a multidisciplinary team, being based on behavior change theory, and a having a design process centered on the user.

The comprehensive evaluation plan includes indicators for the app’s engagement strategies, as well as psychosocial and health outcomes up to 6 months after the birth of a child. This will provide valuable insights into what works for reaching the target group, and will ensure that the findings are transferable and that the data will be broadly relevant to future mobile health interventions. We expect results from the PIFI study in 2017.

## Abbreviations

FIFI: Fathers Infant Feeding Initiative
MARS: Mobile Application Rating Scale
PIFI: Parent Infant Feeding Initiative
RCT: randomized controlled trial
SCT: social cognitive theory
